# Synthesis and Characterisation of Nanocomposite Mo-Fe-B Thin Films Deposited by Magnetron Sputtering

**DOI:** 10.3390/ma14071739

**Published:** 2021-04-01

**Authors:** Paulius Malinovskis, Stefan Fritze, Justinas Palisaitis, Erik Lewin, Jörg Patscheider, Per O. Å. Persson, Ulf Jansson

**Affiliations:** 1 Department of Chemistry-Ångström, Uppsala University, SE-751 21 Uppsala, Sweden; stefan.fritze@kemi.uu.se (S.F.); erik.lewin@kemi.uu.se (E.L.); ulf.jansson@kemi.uu.se (U.J.); 2 Department of Physics, Chemistry, and Biology (IFM), Thin Film Physics Division, Linköping University, SE-581 83 Linköping, Sweden; justinas.palisaitis@liu.se (J.P.); per.persson@liu.se (P.O.Å.P.); 3 Evatec AG, Hauptstrasse 1a, 9477 Trübbach, Switzerland; joerg.patscheider@evatecnet.com

**Keywords:** thin films, magnetron sputtering, Mo-Fe-B, iron, hardness, mechanical properties, amorphous, two amorphous phases, TEM, EELS

## Abstract

Several ternary phases are known in the Mo-Fe-B system. Previous ab initio calculations have predicted that they should exhibit a tempting mix of mechanical and magnetic properties. In this study, we have deposited Mo-Fe-B films with a Fe-content varying from 0–37 at.% using non-reactive DC (direct current) magnetron sputtering. The phase composition, microstructure, and mechanical properties were investigated using X-ray diffraction, scanning transmission electron microscopy, and nanoindentation measurements. Films deposited at 300 °C and with >7 at.% Fe are nanocomposites consisting of two amorphous phases: a metal-rich phase and a metal-deficient phase. Hardness and elastic modulus were reduced with increasing Fe-content from ~29 to ~19 GPa and ~526 to ~353 GPa, respectively. These values result in H^3^/E^2^ ratios of 0.089–0.052 GPa, thereby indicating brittle behaviour of the films. Also, no indication of crystalline ternary phases was observed at temperatures up to 600 °C, suggesting that higher temperatures are required for such films to form.

## 1. Introduction

Transition metal borides (TMBs) can combine attractive properties such as high hardness [1], low friction [2], high electrical conductivity, oxidation resistance [3,4,5], and high corrosion resistance [6]. The brittle behaviour of TMBs is, however, currently a major drawback. Non-reactive magnetron sputtering of binary metal borides is a very efficient method to synthesise well-defined films, where the material properties can be controlled by fine tuning of the experimental process parameters [7]. An excellent example of a magnetron sputtered thin film TMB is TiB_2_, which has been synthesised using both compound and elemental targets. Mayrhofer et al. reported hardness values of 60 GPa for sputtered TiB_2_, which is more than twice as hard than the reported bulk values [1]. The superhardness was attributed to a boron tissue phase that surrounded the grains. Similar hardening with a combination of a nanostructured boride and a boron-rich tissue phase has also been observed for NbB_2_ [2] and MoB_2_ [8].

The structure of the diborides is strongly dependent on the transition metal, and several structures have been reported. The AlB_2_-type structure (space group 191—P6/mmm) is the most stable diboride for group 4 transition metals [5], while the W_2_B_5−x_-type structure (space group 194—P6_3_/mmc) and the hR18-MoB_2_ type structure (space group 166—R-3mh) are reported for group 6 transition metals [8,9,10]. The instability of the AlB_2_-type structure for group 6 transition metals is caused by filling of antibonding orbitals [11,12]. This is illustrated in the Mo-B phase diagram, which shows that the AlB_2_-type is a high temperature phase with a substoichiometric composition and a B/Mo ratio <67 at.%. At lower temperatures (<1517 °C), the hR18-MoB_2_ phase is the thermodynamically most stable phase. Recent reports, however, show that magnetron sputtered WB_2_ [13] and MoB_2_ [8] thin films always crystallise in an AlB_2_-type structure at low deposition temperatures. This can be explained by kinetic reasons, since the AlB_2_ structure is simple with alternating planar metal and B layers. In contrast, the Mo_2_B_5−x_ structure is much more complex with a large unit cell (c-axis about 21 Å) and alternating puckered and planar B-layers [14]. The formation of such a phase requires a significant surface diffusion present.

A major advantage of AlB_2_-type structured MoB_2_ and WB_2_ is that theoretical calculations predict ductile behaviour (Frantsevich criterion) for these materials [10]. A recently introduced design strategy is to add a second metal to the film, in order to enhance mechanical properties [15,16]. Such ternary M_1_-M_2_-B_2_ thin films have only been realized to a limited extent with magnetron sputtering [6,17]. An excellent example is the superhard (46 GPa) Ti-Zr-B_2_ [18]. Enhanced mechanical properties, such as superhardness and high fracture toughness, have been reported for W-M-B_2_ (M = Ta or Ti) thin films [17,19]. A new approach is to alloy MoB_2_ with iron, which cannot only give excellent mechanical properties but also magnetic properties. Furthermore, the Mo-Fe-B system includes several ternary phases such as Mo_2_FeB_2_ and MoFeB_4_. Recent DFT (density functional theory) calculations have demonstrated that also these phases can exhibit an attractive combination of high hardness and ductility [20,21].

The aim of this study was to investigate the influence of Fe on magnetron sputtered MoB_2_ films. Our primary goal has been to investigate if it is possible to deposit thin films of the ternary phases or if Fe can be dissolved into MoB_2_ with the AlB_2_-type structure. A series of Mo-Fe-B films with different Fe contents was therefore deposited by magnetron sputtering using compound MoB_2_ and elemental Fe targets. The correlation between microstructure, crystallinity, and mechanical properties of the magnetron-sputtered Mo-Fe-B films has been studied as a function of Fe content using X-ray diffraction (XRD) and scanning transmission electron microscopy (STEM). The binary Mo-B system has been investigated in an earlier study [8] and is used as a reference for the ternary Mo-Fe-B films.

## 2. Materials and Methods

The Mo-Fe-B films were deposited by non-reactive DC-magnetron sputtering (home-built) equipped with Kurt. J. Lesker magnetrons from MoB_2_ (99.5% claimed purity) and Fe (99.5% claimed purity, specially shaped with groove to maintain the plasma in the groove) 2 inch targets in an ultra-high vacuum chamber (base pressure ~10^−7^ Pa). An Ar^+^ plasma was ignited at 0.4 Pa and 42 sccm Ar gas flow. The MoB_2_ target current was kept constant at 150 mA, while the Fe target current was varied between 0–75 mA. The Si (001) substrates were preheated for 60 min to 300 °C, and a DC bias voltage of −50 V was applied to the substrate table during deposition. An adhesion layer of ca 15 nm Nb was used.

XRD measurements were performed on a Philips X’Pert MRD diffractometer (Almelo, Overijssel, Netherlands) using Cu K_α_ radiation and parallel beam geometry. The grazing incidence XRD (GI-XRD) was performed at a fixed incident angle of 2°. Peak positions and widths were determined by curve fitting of the peaks. The chemical composition in the films was analysed with XPS (X-ray photoelectron spectroscopy) using a Physical Electronics Quantum 2000 (Physical Electronics, Eden Prairie, MN, USA) with monochromatic Al K_α_ radiation and a 45° photoelectron take-off angle. High-resolution spectra were acquired after sputter-etching (1 keV, 10 min, ~40 nm removed) to analyse the bulk of the coating material, below the surface-oxidised region. Compositional and chemical bonding analysis was carried out on coatings on Si (001) substrates. The sensitivity factors were determined from well-defined film compositions determined with Time-of-Flight Energy Elastic Recoil Detection Analysis (ToF-E-ERDA). These experiments were performed at the Tandem Accelerator Laboratory at Uppsala University (Uppsala, Sweden) in a vacuum chamber at a base pressure of <10^−6^ Torr with 36 MeV ^127^I^8+^ ions as primary projectiles. High-resolution (HR)TEM (transmission electron microscopy) and scanning TEM (STEM) combined with high angle annular dark field imaging (STEM-HAADF), selective area electron diffraction (SAED), energy dispersive X-ray spectroscopy (EDX), and electron energy loss spectroscopy (EELS) analysis was performed in the double-corrected Linköping FEI Titan^3^ 60–300 (FEI Company, Hillsboro, OR, USA), operated at 300 kV. Cross-sectional samples were prepared by traditional methods including mechanical grinding and 5–3kV Ar^+^ ion polishing (gradually reduced to minimise the damage).

Mechanical properties (hardness and Young’s modulus) were measured on a CSM Instruments Ultra Nano Hardness Tester (Anton Paar GmbH, Peseux, Switzerland) equipped with a diamond Berkovich tip. Load-displacement curves were measured in 20 different spots. Hardness and elastic modulus were determined following the Oliver-Pharr method and averaged from at least 15 indentation spots [22]. WYKO NT1100 optical profilometer (Veeco, Tucson, AZ, USA) was used to measure the surface curvature and estimate the stress in the film, using Stoney’s equation [23].

## 3. Results and Discussion

A series of Mo-Fe-B films (Table 1) with a Fe content ranging from 0–37 at.% was deposited at 300 °C. The composition of the binary Mo-B film was MoB_1.65_ corresponding to 62 at.% B in the film, which is in good agreement with the composition (61 at.% B content) of the MoB_2_ sputter target. The addition of Fe led to a fluctuation of B/Mo ratio between 1.58–1.68. The films are clearly substoichiometric with the B/(Mo + Fe) ratio <2.

**Figure 1:**
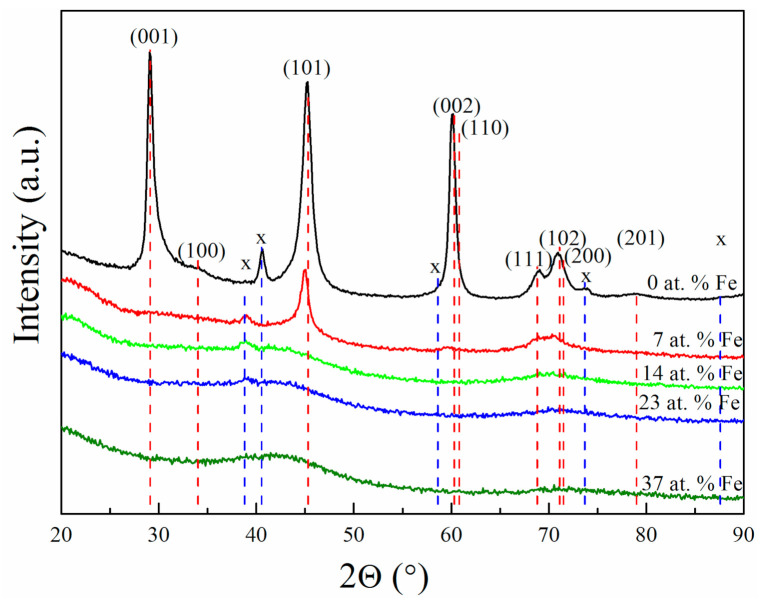
GI-XRD scans on a series of Mo-Fe-B samples with increasing Fe content (0–37 at. %). Miller indices and red-dashed lines indicate the hexagonal MoB_2_ phase [23], “x” and blue dashed lines indicate peaks from the Nb adhesion layer.

Figure 1 shows GI-XRD diffractograms obtained from the deposited films. The binary film (0 at.% Fe) exhibits sharp diffraction peaks from the MoB_2_ phase with a hexagonal AlB_2_ structure [24]. The lattice parameters were determined as a = 3.05 Å and c = 3.07 Å. The addition of 7 at.% Fe leads to an expansion of the unit cell volume from 24.73 Å^3^ to 25.30 Å^3^. Fe has a smaller atomic radius than Mo, and therefore the volume expansion is an indication that the Fe atoms occupy interstitial sites in the MoB_2-x_ structure. Furthermore, alloying MoB_2-x_ with Fe led to a significant reduction of crystallinity (increased width and reduced intensity of peaks), and films with >7 at.% Fe are X-ray amorphous. No indication of a crystalline ternary Mo-Fe-B phase can be observed. An additional experiment was carried out at 600 °C (not shown) to investigate if a higher deposition temperature could promote the formation of ternary phases. No indication of crystalline ternary phases, however, was observed at this temperature. Possibly, much higher temperatures are required to form, for example, the Mo_2_FeB_2_ phase [25].

**Figure 2:**
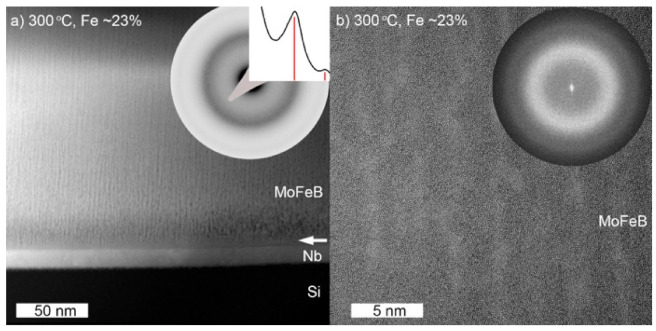
(**a**) Overview cross-sectional STEM-HAADF and corresponding SAED patterns together with radially integrated SAED intensities obtained from Mo-Fe-B samples with 23 at.% of Fe. Arrow indicates the start of columnar structure in the Mo-Fe-B film. (**b**) Cross-section HRTEM images and Fast Fourier Transform (FFT) patterns obtained from the same film.

The cross-sectional STEM-HAADF image and the SAED pattern recorded from the X-ray amorphous film with 23 at.% Fe are shown in Figure 2a. The SAED and radially averaged SAED intensity, shown in the insets of Figure 2a, together with the HRTEM image in Figure 2b, confirms the amorphous microstructure. The formation of amorphous material can be expected since many metal-metalloid glasses are based on Fe and/or B [26,27,28] and is also in agreement with earlier studies on electroplated and magnetron-sputtered Mo-Fe-B films [21,29]. Furthermore, the formation of a metallic glass-like microstructure is also favored by the fact that the crystal structures of the ternary phases in the Mo-Fe-B system are rather complex, which means that a significant amount of diffusion and redistribution of all elements are required during the deposition process to form a multiphase material with crystalline grains. DFT calculations by Dahlqvist et al. have recently shown that MoB_2-x_ is stabilised by vacancies on the boron sublattice [30]. The addition of Fe, which has a higher number of valence electrons compared to Mo, should therefore increase the number of states at the Fermi level and lead to a destabilization of MoB_2-x_ phase and favor the formation of an amorphous structure.

**Figure 3:**
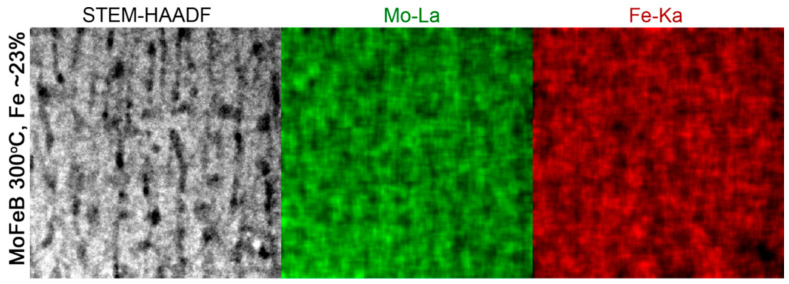
Cross-sectional STEM-HAADF images and corresponding EDX elemental maps displaying the distribution of Mo and Fe in Mo-Fe-B film with 23 at.% of Fe. The images are 40 × 40 nm.

The STEM-HAADF image was acquired using strong elemental contrast (Z-contrast) conditions and suggests that two phases are present in the film. Both phases are stretched in growth direction throughout the film thickness, giving rise to a columnar appearance. Figure 2b clearly shows two columnar features, one darker and one lighter, which can be attributed to two amorphous phases of different composition. The elemental constituents of the two phases were examined using STEM-EDX/EELS. The STEM-EDX maps revealed that the bright contrast areas in the film are Mo-Fe enriched, while darker areas are metal-deficient (Figure 3).

**Figure 4:**
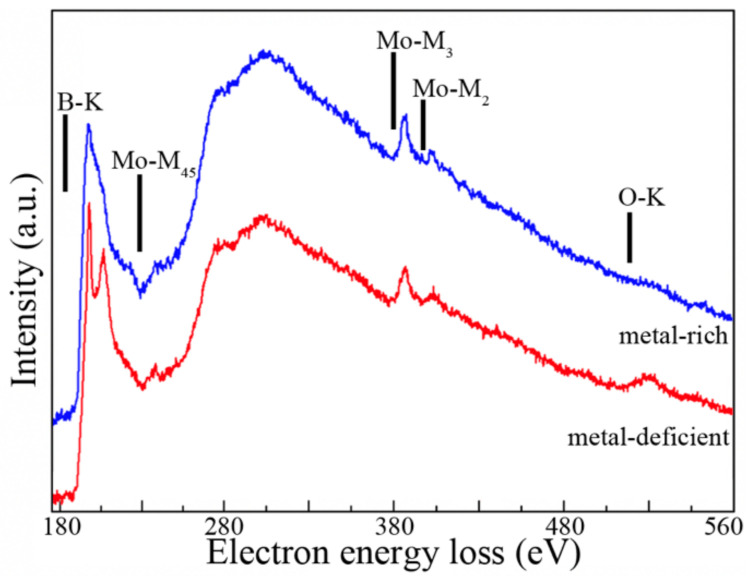
Core-loss EELS spectra recorded from metal-rich and metal-deficient amorphous phases in Mo-Fe-B film with 23 at.% of Fe grown at 300 °C. The spectra are normalized with respect to the integrated signal and laterally displaced for a better presentation.

Furthermore, STEM-EELS spectra (see Figure 4) revealed the presence of a pronounced O-K edge intensity, indicating a higher oxygen content together with a distinct difference in the B chemical environment, in the metal-deficient phase compared to the metal-rich phase. A phase separation into two amorphous phases is expected, since most metallic glasses have the best glass-forming ability at low and moderate concentrations of p-elements. It can thus be anticipated that a metal-rich amorphous phase grows in columnar-like structure separated by a metal-deficient amorphous phase.

Nanoindentation measurements were performed to determine the hardness and elastic moduli of the films; the results are summarised in Table 1. The hardness decreases from 29 ± 2 GPa for the binary film to 18.7 ± 1 GPa for the film with 37 at.% Fe. The elastic modulus shows a similar trend and is reduced from 526 GPa to 353 GPa with increasing Fe content. The lower hardness of the amorphous films is caused by the change of deformation mechanism between crystalline (dislocation movement) and amorphous materials (deformation via shear bands). The lower Young’s modulus is a result of changed bonding states in the films. However, a maximum hardness of 22 GPa for the amorphous films is about 4 GPa higher than reported values for amorphous phases in the Nb-Fe-B system [31]. The increased hardness in the Mo-Fe-B films is a result of a more Mo-rich composition compared to the Fe-rich compositions in ref. [31]. The binary Mo-B film exhibits compressive stress of 0.1 GPa, and all Fe-containing films exhibit compressive stresses in the range of 0.03 GPa to 0.4 GPa, which suggests that the hardness values are not significantly influenced by stresses in the films. All films exhibit H^3^/E^2^ values ranging from 0.089–0.052 GPa, indicating that the ductility is not significantly affected by Fe introduction [32].

## 4. Conclusions

The major aim of this study was to investigate the possibilities to deposit ternary films in the Mo-Fe-B system. Previous DFT calculations suggest that such films could exhibit unique mechanical and magnetic properties and therefore have potential use in thin film applications. Our results, however, showed that no such ternary crystalline phases were formed although the temperature was increased to 600 °C. This suggests that even higher temperatures are required to form these rather complex crystalline structures with large unit cells. Potentially, the formation of ternary phases could be favoured using alternative deposition methods such as high power impulse magnetron sputtering (HiPIMS).

The maximum solid solution of Fe into the hexagonal MoB_2_ phase is low and X-ray amorphous films were observed above 7 at.% Fe. The formation of an amorphous phase upon the addition of Fe is not unexpected since previous DFT calculations have showed that an increase in valence electron concentration reduces the stability of the diboride phase. The deposited non-crystalline films showed an interesting multiphase mixture of two separate amorphous phases. The properties of such multiphase amorphous films are less well studied in the literature and are an interesting area for future studies.

## Figures and Tables

**Table 1:** Chemical composition (obtained by ERDA and XPS) and mechanical properties (obtained by nanoindentation) of Mo-Fe-B films deposited with different Fe target currents.

Fe Target Current (mA)	Composition (at.%)				B/Mo Ratio	H (GPa)	E (GPa)
	Mo	B	Fe	O			
0	37	62	-	≤2	1.65	29.1 ± 1.9	526 ± 26
12	34	58	7	≤1	1.68	22.2 ± 0.8	422 ± 10
25	30	55	14	≤1	1.68	21.2 ± 1.0	395 ± 19
40	27	49	23	≤1	1.62	20. 8± 1.0	377 ± 15
75	23	39	37	≤1	1.58	18.7 ± 0.6	353 ± 11

## Data Availability

Not Applicable.
